# Some Remarks on Cardiac Hypertrophy and Dilatation

**Published:** 1859-11

**Authors:** Austin Flint

**Affiliations:** Professor of Clinical Medicine, etc., in the New Orleans School of Medicine


II.—*Some Remarks on Cardiac Hypertrophy and Dilatation.* By AUSTIN FLINT, M.D., Professor of Clinical Medicine, etc., in the New Orleans School of Medicine.

CORRECT views respecting the two forms of enlargement of the heart—hypertrophy and dilatation—especially as regards the abnormal circumstances under which they are produced, their relations to each other, and the effects of each, are important on account of their direct practical bearings. The clinical study of cardiac diseases has lately led to a complete reversal of certain ideas connected with hypertrophy; and the consequence is, a reversal of the practice which, until quite recently, the existence of this form of enlargement was supposed to require. But there is reason to believe that this change is not so generally known or so fully appreciated by practitioners of medicine as, in view of its importance, is to be desired. This consideration mainly has determined the selection of the subject of this article. I shall endeavor in a few remarks to embody a concise expression of practical views which it is believed are in accordance with sound pathology, as well as with the conclusions drawn from clinical experience, and which, if not in some respects original, have not, as it seems to me, been fully presented, and their application to practice sufficiently considered.

Hypertrophy and dilatation are effects of an obvious mechanical obstruction to the circulation, in the great majority of cases. It is probable that in the comparatively few instances in which an obstacle is not apparent, it nevertheless exists. This remark is, however, more applicable to hypertrophy than to dilatation. Practically, it may be assumed that if the heart be hypertrophied, there either is, or has been, over-accumulation within the cavities of the heart from some impediment to the free passage of the blood either through the cardiac orifices or the vessels. In by far the greater proportion of cases the obstacle arises from valvular lesions, which occasion either obstruction or regurgitation, or both; and it will suffice for the present purpose to limit the attention to hypertrophy and dilatation occurring as consequences of these lesions.

Valvular lesions give rise to hypertrophy and dilatation, which, when death results from disease of the heart, are usually found to be combined in variable proportions. It is rare to find hypertrophy without dilatation, or dilatation without hypertrophy. Both forms of enlargement are alike consequences as valvular lesions, but the latter do not give rise indifferently to the one or the other form. What are the laws which govern their mutual relations? In answer to this question the following may be stated, as deductions from clinical facts: Hypertrophy is uniformly the first form of enlargement produced as a consequence of an obstacle to the circulation, provided the muscular structure of the heart be unaltered and its vigor unimpaired. Hypertrophy, under these circumstances, precedes dilatation. The two forms of enlargement do not, as stated by some writers, go on together from the commencement, but dilatation begins after hypertrophy has been going on for an indefinite period. In the course of time, dilatation is as inevitable as the precedence of hypertrophy is invariable. These laws hold good except in the cases in which the walls of the heart are the seat of fatty degeneration, or other changes, or the muscular power of the organ from any cause has been permanently impaired. The following are clinical facts leading to the foregoing deductions: In persons affected with valvular lesions, together with more or less cardiac enlargement, who are cut off by some intercurrent or incidental affection before suffering from any marked symptoms referable directly to the heart, examination after death shows hypertrophy either existing without dilatation, or greatly predominating over the latter. *Per contra*, in persons who die after a protracted duration of disease of the heart, having presented before death characteristic symptoms, such as dyspnœa, lividity, and general dropsy, dilatation is found to predominate over hypertrophy.

The production of hypertrophy, in view of the obstacle which occasions it, is compensatory. In health the vigorous contractions of the ventricles are complete; that is, the blood is entirely expelled, leaving the ventricular cavities void at the close of the systole. It is not easy to demonstrate this, but proof was afforded in a case falling under my observation of mitral lesions, in which a small mass of calcareous deposit projected from the ventricular aspect of the anterior curtain of the valve. Directly opposite to this mass, the endocardial membrane, over a space corresponding in size, was opake and thickened,—in fact, calloused, the membrane surrounding this spot being perfectly normal. The callosity was obviously due to long-continued repetitions of forcible contact with the calcareous mass, thus showing that the endocardial surfaces are brought into apposition by the systolic contractions. For this completeness of the ventricular contractions to continue when an abnormal obstacle to the circulation exists—for example, let it be supposed that the aortic orifice is contracted—the power of the contractions must be increased proportionally to the impediment caused by the contraction of the orifice. This increased power is a result of the obstacle to the egress of blood through the aorta, and the consequence is, increased muscular growth, or hypertrophy of the left ventricle. This increased growth imparts to the ventricle a greater amount of power, so that its capability to compensate for an increasing difficulty is extended in proportion to the progress of the hypertrophy, the latter, conversely, keeping pace with the increase of the obstacle due to the progressive obstruction at the orifice of the aorta. The same results follow when lesions, either obstructive or regurgitant, are situated at the mitral, tricuspid, or pulmonic orifices, the difference being chiefly as regards the ventricle first affected. The laws are, *first*, augmentation of muscular power compensating for the obstacle, or proportionate to the undue accumulation of blood in one or both of the ventricular cavities; *second*, increased muscular growth or hypertrophy; *third*, greater capability of exerting muscular power, proportionate to the amount of hypertrophic enlargement.

It is evident from this view of the immediate pathological consequences of valvular lesions, that hypertrophy is a conservative provision designed to obviate ulterior morbid effects. This idea, however, has but recently been appreciated, or, at all events, it is only of late that it has began to exert its legitimate influence on practice. Practitioners have aimed to diminish the hypertrophy, or prevent its further progress; and for this end potent measures have been resorted to, viz., copious blood-lettings and other methods of depletion, low diet, and as much quietude as possible. So far from these objects of treatment being desirable, they conflict directly with conditions on which the comfort and safety of the patient depend. The practitioner should strive rather to maintain the hypertrophy, and to govern its increase in proportion to the increasing impediment to the circulation due to progressive valvular lesions. The existence of hypertrophy does not call for measures to lower the powers of the system and weaken the heart, but, on the contrary, the body should be well nourished, and the vigorous action of the ventricles promoted.

Not only are depletory and debilitating measures uncalled for by the hypertrophy, but an opposite plan of treatment is indicated, viz., a good diet, tonic remedies, and exercise so far as it can be taken without a sense of discomfort. It is somewhat difficult at once to receive practical views diametrically at variance with those which have hitherto guided medical practice, under the sanction of high authority; but clinical observation, as well as sound pathology, shows the importance of hypertrophy as a conservative provision against the secondary and remote evils arising from valvular lesions. As a rule, these lesions, so far as symptoms are concerned, are latent while the impediment to the circulation which they occasion is compensated for by the muscular growth of the heart. This period of latency often lasts for many years. Persons experience no inconvenience from symptoms referable to the heart, leading them to be apprehensive of cardiac disease. They do not go to the physician with ailments dependent on the condition of the heart for a long time after the lesions of the valves have induced hypertrophy. The existence of cardiac disease at this stage is often ascertained incidentally, patients coming under observation with various affections which have no direct connection with the heart. Moreover, of persons affected with valvular lesions and hypertrophy, they have remained for the longest period exempt from distressing symptoms referable to the cardiac disease, who have continued to live well and take abundant exercise, not being fully aware of their condition, and not subjected to debilitating medication. My own observations furnish a number of instances strikingly illustrative of this remark. Numerous cases have been reported by Dr. Stokes and others. In fine, the alternative being between hypertrophy and dilatation, in cases of valvular lesions which by obstruction or regurgitation occasion an impediment to the circulation through the heart, the former is not merely to be preferred as a choice of evils, but it is highly important as a protection against the latter.

Hypertrophous enlargement of muscular organs has its bounds. Having attained to a certain size, their further increase is impossible. The heart, like the voluntary muscles, in addition to its normal extent of development, has a capacity for only a definite amount of abnormal growth. The restriction to hypertrophy is inherent in the muscular tissue. Extrinsic circumstances cannot carry the growth beyond a fixed point. On what does this intrinsic limitation depend? This inquiry cannot be answered without more knowledge of the mechanism of muscular growth than has as yet been acquired. It appears not to be settled demonstratively whether the muscles increase in size by the formation of new fibrils or by the enlargement of those already formed. In the one case, hypertrophy involves a hypergenesis of the structural elements; in the other case, the process is simply exaggerated nutrition. The former is to be inferred from the extent of the capacity of the muscles for hypertrophous enlargement. This intrinsic capacity, as regards the heart, probably varies in different persons. The limits of hypertrophy having been reached, and the impediment to the circulation persisting, dilatation necessarily follows. So soon as the muscular power of the ventricles is inadequate to completeness of contraction, and the walls of the heart meet with a resistance from the blood accumulated within the cavities, which they cannot overcome, dilatation becomes a necessity. From this epoch dilatation goes on with a rapidity, other things being equal, proportionate to the distention of the ventricles from over-accumulation. In this way dilatation becomes combined with hypertrophy, and the dilatation is more or less predominant according to the degree of impediment to the circulation, and the duration of life after the hypertrophous growth has ceased. When the latter has reached its utmost bounds, and existence has been prolonged, so that the dilatation is extreme, the heart attains to an enormous size, constituting the *cor bovinum* of the older writers. The pathological processes involved in the production of hypertrophy on the one hand, and on the other hand of dilatation, it will be observed, are quite different. In hypergenesis and hypernutrition the processes are vital, but distention and dilatation are processes purely mechanical. The former result from the exaggerated power of a vital function, viz., muscular contraction; the latter proceed from passive yielding of the tissues.

In this sketch of the production, successively, of hypertrophy and dilatation, it is assumed that the muscular structure of the heart is unchanged, and that morbid weakness of the organ does not exist. If the walls of the ventricles have undergone extensive fatty degeneration, for example, the rule of the succession of hypertrophy and dilatation may not hold good. A vital reaction sufficient to overcome the increased resistance is not called forth under the circumstances. The hypertrophous growth is slight or wanting. Yielding of the ventricular walls takes place speedily or at once, and dilatation thus occurs, with little or no hypertrophy. Weakness of the heart, induced from any cause, for example injudicious treatment by blood-letting and other debilitating measures, may cause an arrest of the hypertrophous growth before it has reached its intrinsic bounds, and in this way is hastened the epoch when the enlargement by dilatation commences. If the heart be greatly weakened by organic change, (fatty degeneration,) the ventricles may yield to the distention occasioned by the accumulation of the blood in the cavities, without any impediment to the circulation from valvular lesions or other obstacles except the diminished muscular power of the organ. Clinical experience furnishes examples of dilatation resulting exclusively from the latter cause.

And according to the foregoing statements, if, in connection with valvular lesions, dilatation be found after death to exist either without or with only a slight degree of hypertrophy, we should expect to find the muscular structure altered. In other words, in cases of enlargement of the heart proceeding from valvular lesions, if hypertrophy exist alone, or in a marked degree in connection with dilatation, we should expect to find the muscular structure normal, save as regards the morbid growth. Clinical experience verifies the correctness of these conclusions.

Of the two forms of enlargement of the heart, dilatation is the form to be dreaded. The production of hypertrophy, as has been seen, for a certain period compensates for an existing impediment to the circulation, and is a conservative provision. It is otherwise with dilatation. This is evidence that the ability to overcome an abnormal resistance no longer exists. The organ now begins to give way, and having once began to yield, the dilatation goes on progressively, with more or less rapidity, according to various circumstances, intrinsic and extrinsic. After a time the dilatation predominates over the hypertrophy. This is an important epoch in the progress of cardiac disease. From this period distressing symptomatic events referable to the cardiac disease are liable to occur, viz., dyspnœa, orthopnœa, and general dropsy. Prior to this epoch, although the hypertrophy and dilatation have existed and been gradually increasing for many months, or even years, the patient may not have experienced symptoms which led him to suspect cardiac trouble. It is at this epoch that frequently cases for the first time come under medical observation. The evils and danger connected with the disease arise mainly from the weakness and incompleteness of the heart's contractions. The cavities are constantly over-distended, and as the muscular power of the organ diminishes more and more, the over-accumulation of blood, and consequent dilatation, progressively increase. Death, not attributable to an incidental occurrence or an intercurrent affection, but due directly and exclusively to the cardiac disease, takes place in consequence of over-distention and a loss of muscular power amounting to paralysis. When death is in this way produced, it occurs after a prolonged period of intense suffering. Happily, (we may almost say,) a fatal result as thus produced, in a majority of cases, is anticipated by some one or more of various contingencies and accidental events, such as the formation of clots in the heart, œdema of the lungs, pleuritic effusion, apoplexy, etc. etc.

Regarding the evils and danger of dilatation, as well as the compensatory and conservative character of hypertrophy, and it is sufficiently obvious that the objects of treatment should be to maintain the former and postpone the latter. Hence it is clear that measures relating either to diet, exercise, or medication, which weaken the muscular power of the heart, must prove hurtful in proportion to their potency, since they conflict directly with the objects just stated. That the practice of treating patients affected with valvular lesions and enlargement by blood-letting, low diet, and extreme quietude, has been mischievous, is not less intelligible on rational grounds than evidenced by clinical experience. The objects of treatment which have been stated relate to any period in the progress of the disease anterior to the time when the dilatation predominates. When this time arrives, the recovery of predominating hypertrophy is impossible, and the object of treatment which remains, is to retard, as much as possible, the increase of dilatation, by measures which tend to support the muscular vigor which the heart still retains.

